# Prediction of post-operative pain following arthroscopic subacromial decompression surgery: an observational study

**DOI:** 10.12688/f1000research.2-31.v1

**Published:** 2013-02-04

**Authors:** Anthony Davis, David J Chinn, Sunil Sharma

**Affiliations:** 1 Anesthesiology Department, NHS Fife, Victoria Hospital, Kirkcaldy, Fife, KY2 5AH, UK; 2 Research and Development, NHS Fife, Lynebank Hospital, Dunfermline, Fife, KY11 4UW, UK; 3 Orthopaedics, NHS Fife, Victoria Hospital, Kirkcaldy, Fife, KY2 5AH, UK

## Abstract

**Background**: Arthroscopic shoulder surgery is increasingly performed as a day case procedure. Optimal post-operative pain relief remains a challenge due to considerable variations in the level of pain experienced between individuals. Our aim was to examine whether the preoperative electrical pain threshold was a strong predictor of elevated postoperative pain levels following arthroscopic subacromial decompression (ASD) surgery. **Methods:** Forty consenting patients with American Society of Anesthesiologists (ASA) grade 1-2 presenting for elective ASD surgery were recruited. Patients’ electrical pain thresholds were measured preoperatively using a PainMatcher® (Cefar Medical AB, Lund, Sweden) device. Following surgery under general anaesthesia, the maximum pain experienced at rest and movement was recorded using a visual analogue scale until the end of postoperative day four. **Results**: In univariate analyses (t-test), the postoperative pain experienced (Area Under Curve) was significantly greater in patients with a low pain threshold as compared with a high pain threshold at both rest (mean 12.5, S.E. 1.7 v mean 6.5, S.E.1.2. P=0.008) and on movement (mean 18.7, S.E. 1.5 v mean 14.1, S.E.1.4. P=0.031). In multivariate analyses, adjusting for additional extra analgesia, the pain experienced postoperatively was significantly greater in the low pain threshold group both at rest (mean difference 4.9, 95% CI 1.5 to 8.4, P=0.007) and on movement (mean difference 4.1, 95%CI 0.03 to 8.2, P=0.049). **Conclusions**: Preoperative pain threshold can predict postoperative pain level following ASD of the shoulder. **Trial registration**: Clinicaltrials.gov identifier: NCT01351363 **Level of Evidence**: II

## Introduction

Increasingly, arthroscopic shoulder surgery is being performed as a day case procedure. In this setting, optimal post-operative pain relief is the goal. This not only improves patient comfort and allows expedient discharge from hospital, but also reduces the risk of developing post-operative chronic pain and may improve surgical outcome ^1– 4^.

Optimal post-operative pain control in day-case surgery remains a challenge ^5^. There is considerable variation in the level of post-operative pain experienced between individuals and subsequent analgesia requirements. Previous studies have attempted to predict the level of acute post-operative pain an individual will experience, using a variety of complex pre-operative pain and psychological assessments ^6, 7^. Other investigators have focussed on a simpler approach, by testing an individual’s pain threshold to a single pre-operative nociceptive stimulus, e.g. heat or pressure ^8– 11^. An increase in pain sensitivity in these pre-operative experimental tests appears to correlate with a higher level of post-operative pain and an increased risk of developing persistent post-operative pain ^12^. Electrocutaneous stimulation has also been shown to be a reliable and safe method for assessment of pain and sensory thresholds ^6, 13– 18^. The technique has previously been used as a predictive tool in the Obstetric surgery setting using the Pain Matcher ^®^ (Cefar Medical AB, Lund, Sweden) ^19^.

The Pain Matcher is a small hand held device that has been validated against the visual analogue scale (VAS) for reliable pain assessment in patients with a range of acute and chronic pain, as well as for pre-operative pain threshold assessment ^19– 24^. When the contact pads on the Pain Matcher are gripped between the thumb and forefinger, the device delivers a small, micro-processor controlled, non-harmful electric current to the individual. Variations between individuals’ skin resistance are compensated for. The electrical current consists of rectangular pulses at 10 Hz with 10mA amplitude, which is gradually increased by 4 μs rises in pulse width, from zero to a maximum of 396 μs. This occurs over a total of 99 steps and ceases when the individual releases their grip on the device. The results are displayed on an LCD screen, on a scale from 1 to 99, which is directly related to the pulse width. Higher Pain Matcher values indicate a higher pain threshold ^17, 18, 21^.

We aimed to assess the predictive value of pre-operative pain threshold measurements, made via electrocutaneous stimulation, for the intensity of post-operative pain experienced following arthroscopic subacromial decompression surgery.

## Materials & methods

Ethical approval for this study (09/S0501/25) was provided by the NHS Fife & Forth Valley Research Ethics Committee, Dundee, UK (Chairperson Mr G Costa) on 9 ^th^ March 2009.

### Patients

Forty adult patients with American Society of Anesthesiologists (ASA) grade 1–2, presenting for elective day case arthroscopic subacromial decompression surgery at the Victoria Hospital, Kirkcaldy, UK, were recruited into the study between May 2009 and October 2010 at the pre-operative surgical assessment clinic, held one week prior to surgery. Full written consent was obtained from all study participants.

Exclusion criteria were: inability to give informed consent; inability to perform a telephone interview; an allergy to anaesthetic and analgesic drugs used in this study; drug or alcohol abuse; a formal diagnosis of chronic pain; a formal diagnosis of a neurological or psychiatric disorder; the use of neuromodulatory drugs, or daily analgesics; documented sensory abnormality (eg. peripheral neuropathy); or a documented rotator cuff tear.

### Pre-operative pain threshold testing

Patients were tested on the day of surgery following a six hour period of fasting and abstinence from any analgesics. We recorded age, Body Mass Index (BMI), ASA classification and telephone numbers.

Pre-operative electrical pain thresholds (EPT) were recorded using the Pain Matcher device. Patients were asked to grip the device until the stimulus was first experienced as painful. After an initial "practice run" with the Pain Matcher, pre-operative pain thresholds were measured using the patient’s hand contra-lateral to that side undergoing surgery.

### Anaesthesia and surgery

Anaesthesia was conducted by a single anaesthetist blinded to the patient’s EPT. Following application of standard monitoring, all patients received target controlled total intravenous anaesthesia (Orchestra, Fresenius-Kabi AG, Germany) using propofol (Marsh protocol; effect site concentration 3.5–7 μg/ml) and remifentanil (Minto protocol; effect site concentration 4–7 ng/ml) for both induction and maintenance of anaesthesia. Intermittent positive pressure ventilation was via a laryngeal mask airway, using oxygen and air (FiO _2_ 50%). In addition, all patients received ondansetron 4mg, glycopyrrolate 200 μg and morphine 0.1mg/kg. All patients received an arthroscopic subacromial decompression, performed by a single orthopaedic surgeon, who also administered a subacromial bursa injection of 30ml 0.5% levobupivacaine (Abbott Laboratories, UK) at the commencement of surgery.

### Postoperative analgesia

Upon recovery from anaesthesia, patients were discharged from the theatre recovery suite once VAS pain scores (0–10) were less than 4. Intravenous morphine was administered to local protocols until this pain score was achieved. Additional rescue analgesia (oral tramadol 100mg) was available on the day case ward prior to discharge.

Patients were discharged home on the day of surgery. Outpatient analgesia consisted of paracetamol 1g 6 hourly, codeine phosphate 60mg 6 hourly and diclofenac 50mg 8 hourly. Additional analgesia, if required, was available from the hospital or the patient’s General Practitioner.

### Follow-up interview

Patients were introduced to the use of a VAS pre-operatively and asked to record the worst pain experienced at rest and at movement for the first four postoperative days. Telephone numbers were obtained to allow the VAS scores to be obtained via a telephone interview on postoperative day five.

The highest pain score experienced at rest and on movement in each 24 hour period was recorded. The use of additional analgesics was also recorded.

### Statistical analysis

Data were analysed with parametric and non-parametric tests using SPSS. The post-operative daily pain scores were used to determine an Area under the Curve (AUC) ^23^ which was related to the pre-operative EPT scores in univariate and multivariate (linear regression) analyses allowing for potential confounders. A power analysis suggested we needed about 40 subjects for the multiple regression based on a recommendation of 10 patients per variable included ^20^. A p value of less than 0.05 was accepted as being statistically significant and 95% confidence intervals (CI) were generated where relevant.

## Results

Forty patients meeting the inclusion criteria were initially approached and recruited into the study. No eligible patients declined to enter the study. Data were incomplete for 9 patients, who were removed from the study, leaving 31 patients (16 women, 15 men) ( Table 1). Body mass index was above 30 kg/m ^2^ in 10 patients (6 females).

**Table 1.** Age and Body Mass Index by gender and ASA grade (n=31).

		Age	BMI (kg/m ^2^)
Females (n=16)	Mean (SD)	50 (7.8)	29.2 (5.8)
	Range	42–69	22–42
Males (n=15)	Mean (SD)	52.8 (14.5)	27.4 (4.0)
	Range	20–68	20–35
ASA grade 1 (n=9)	Mean (SD)	44.4 (15.1)	28.3 (5.1)
	Range	20–67	20–36
ASA grade 2 (n=22)	Mean (SD)	54.2 (8.4)	28.4 (5.1)
	Range	42–69	22–42

SD = Standard deviation.

ASA = American Society of Anesthesiologists grading score.

### Preoperative electrical pain thresholds

The median EPT for the total study group (n=31) was 21 (range: 4–99). Median EPT was 30 (range: 4–99) in men and 19 (range: 4–37) in women (P=0.022, Mann-Whitney U test).

### Postoperative pain

No patients required additional morphine or tramadol post-operatively before discharge from hospital. Six patients requested additional analgesia in the first four post-operative days. The median post-operative pain scores and AUC are shown in Table 2.

**Table 2.** Median post-operative pain scores and Area Under the Curve (AUC) at rest and on movement by gender, days 1–4.

*Day*	*Visual analogue pain scores, median (Range)*			
	Males (n=15)		Females (n=16)	
	Rest	Movement	Rest	Movement
1	3.0 (1–8)	6.0 (2–9)	2.0 (0–8)	5.5 (2–9)
2	2.0 (0–8)	5.0 (1–7)	2.0 (0–8)	4.5 (2–8)
3	2.0 (0–6)	4.0 (1–7)	2.5 (0–8)	4.0 (1–8)
4	2.0 (0–4)	4.0 (1–7)	2.5 (0–6)	4.0 (0–6)
AUC	7.0 (1.0–24.0)	18.0 (4.5–24.5)	7.7 (0–27.0)	15.5 (6.5–27.0)

The post-operative pain experienced (AUC) did not differ by gender (P=0.93 at rest and 0.78 on movement) or ASA grade (P=0.27 at rest and 0.31 on movement). Patients using additional analgesics (n=6) experienced significantly higher post-operative pain than those who did not (n=25) at both rest, mean (SE) AUC 17.8 (2.7) vs 7.6 (1.0) P<0.001 and at movement, mean (SE) AUC 20.7 (1.7) vs 15.4 (1.2) P=0.053 (un-matched t-tests).

### Association between preoperative EPT and post-operative pain scores

Due to the significant difference between male and female EPT scores, patients were subdivided into one of two groups depending on whether their EPT was below or above the gender-specific median (men 30 and women 19, respectively). Those below the median were judged to have a lower pain threshold compared to those above. In univariate analyses (t-test), the post-operative pain experienced at rest, as expressed by the AUC, was significantly greater in those with a low pain threshold (low EPT) as compared with a high pain threshold (high EPT) (P=0.008) ( Table 3). The level of post-operative pain experienced on movement was similarly greater in the lower pain threshold (low EPT) group (P=0.031).

**Table 3.** Post-operative pain experienced (Area under the curve, AUC) by low and high pain threshold groups.

			*AUC at rest*				*AUC on movement*			
		n	Mean	SE	95% CI	P	Mean	SE	95% CI	P
Pain	Low*	16	12.5	1.7	–	–	18.7	1.5	–	–
Threshold	High**	15	6.5	1.2	–	–	14.1	1.4	–	–
	Difference		6.0	2.1	1.7, 10.3	0.008	4.6	2.0	0.5, 8.8	0.031

* Below or equal to the gender-specific median Electrical Pain Threshold (EPT).

** Above the gender-specific median EPT.

AUC: Area under the Curve; SE: standard error; CI: confidence interval; P significance level on unmatched t-test.

In multivariate analyses, after adjusting for the requirement for extra analgesia, the pain experienced post-operatively (AUC) was significantly greater in the low pain threshold (low EPT) group both at rest (mean difference 4.9, 95% CI 1.5 to 8.4, P=0.007) and on movement (mean difference 4.1, 95% CI 0.03 to 8.2, P=0.049).

## Discussion

We have shown that use of a simple pre-operative assessment of pain threshold can be used to predict those patients likely to experience a higher intensity of post-operative pain during the first 4 days following arthroscopic subacromial decompression surgery. Patients with a low pain threshold (EPT below/equal to the gender specific median) reported significantly more post-operative pain both at rest and on movement than those with a high pain threshold (EPT above the gender specific median). These results are in agreement with previous investigations in obstetric surgery that demonstrated pre-operative EPT testing can be used to gain information on the likely intensity of post-operative pain ^19^. The relationship we have demonstrated appears to be clearer with post-operative pain at rest than with pain on movement. This may be because our study did not specify a standardized movement for pain assessment. In addition, we are also in agreement with previous studies, including those using other painful stimuli, which show that women have a lower pain threshold than men ^21, 24^.

Our study population, whilst being generally healthy (ASA grade 1 and 2) was, on average, middle aged (mean 51 years) and “overweight” (mean BMI 28.4 kg/m ^3^) with a third of patients having a BMI above 30, and therefore considered obese. We excluded volunteers suffering from conditions that may have influenced the experience of both preoperative and postoperative pain, i.e. chronic pain, heavy analgesic use or any neurological or psychiatric disorder, and only considered one type of arthroscopic shoulder surgery. It is likely that our results may not translate easily to other patient populations and different shoulder pathologies. Further study on these patient population groups is needed.

The Pain Matcher provides a non-harmful, rapid and easy to administer bedside test of pre-operative pain threshold. Our study did not test levels of pre-existing pain nor undertake any psychological testing, both of which are likely to have effects on pain perception. Despite the simplicity of the Pain Matcher pre-operative testing, our results are comparable to those produced by more elaborate studies ^8– 11^. Although our study contained a relatively small sample, the statistical power was sufficient to detect a difference in pain experienced in relation to pre-operative pain thresholds using simple comparisons and multiple regression analyses.

## Conclusion

Pre-operative screening of patients with the Pain Matcher before arthroscopic subacromial decompression surgery may allow prediction of the likely intensity of post-operative pain experienced. It is a rapid and simple bedside test and may become a useful tool for targeting postoperative analgesia regimens. Further investigations are needed to elucidate whether this affects surgical outcome or the incidence of post-operative chronic pain.
