# Supplementary material for: Prediction of post-operative pain following arthroscopic subacromial decompression surgery: an observational study
Source: F1000Res. 2013 Feb 4;2:31. [Version 1] doi: 10.12688/f1000research.2-31.v1 (PMC3790597; doi:10.12688/f1000research.2-31.v1)
Supplement: Peer review history

# Peer review: Referee response for version 1

**Published:** 2013-06-19

**Authors:** Irene Lund

**Affiliations:** 1 Department of Physiology and Pharmacology, Karolinska Institute, Stockholm, Sweden

In order to contribute to a better control of postoperative pain, this is a very interesting, well described and important clinical study. There are, however, some issues to discuss:

- Explanation to why there were incomplete data for 9 patients.

- Description on how the postoperative pain assessments using VAS were performed.

- The rationale for using both parametric and non-parametric tests. In the manuscript the pain data (by the use of VAS and Pain Matcher) are shown as median and range, referring to that the dataset has ordinal properties, which are adequate for subjectively based assessments such as pain thresholds where the assumption of the distribution of the data is not necessary. In this case, the non-parametric analysis will give more meaning to the analysis than the use of parametric analysis that is based on data that have linear properties and that require knowledge of the data material distribution.

- The results are presented separate for women and men in table 1 and 2 which is very interesting since obvious gender differences are reported in threshold assessments. It would have been interesting to see separate results based on gender in table 3.

- The reported values of pain thresholds represent a wide range from 4-99 in men and 4-37 in women. In other studies using the Pain Matcher, the lowest level of your reported pain threshold is reported in the range of the sensory threshold assessed by Pain Matcher. Could the finding be due to the instruction given to the patients on what they were supposed to feel when reaching the pain threshold? In other words, could the sensation of unpleasantness, present before reaching the pain threshold, be a part of the results and thereby explain some of the great dispersion in the data? 

I have read this submission. I believe that I have an appropriate level of expertise to confirm that it is of an acceptable scientific standard, however I have significant reservations, as outlined above.

# Peer review: Referee response for version 1

**Published:** 2013-06-12

**Authors:** Claudia Campbell

**Affiliations:** 1 Department of Psychiatry & Behavioral Sciences, Johns Hopkins University, Baltimore, MD, USA

This is an interesting, well-written and straight-forward study. The analyses appear to be appropriate to the study aims. Some explanation should be given for the exclusion of nine participants and information provided regarding if they are different from the rest of the sample in some way. Additional demographic information would also be of interest. For example, the duration of shoulder pain and pre-operative pain rating would be of value in placing these results in context.

I have read this submission. I believe that I have an appropriate level of expertise to confirm that it is of an acceptable scientific standard.

# Peer review: Referee response for version 1

**Published:** 2013-02-07

**Authors:** Darin Correll

**Affiliations:** 1 Department of Anesthesiology, Perioperative and Pain Medicine, Brigham & Women's Hospital, Boston, MA, USA

This study is both interesting and potentially of great importance to help clinicians in improving postoperative pain management for patients. The issue however is that almost 25% of the patients originally enrolled were not included in the analysis without adequate explanation as to why this was (e.g. were they missing at random or not) nor a discussion of the fact that this may have had a major impact on the outcomes. Even though the 'data were incomplete' for these 9 patients there are methods for dealing with this from a statistical perspective that may allow a (more) complete analysis. The other option would have been to recruit more patients to make up for this loss and still meet (or come closer to) the pre-determined sample size.

I have read this submission. I believe that I have an appropriate level of expertise to confirm that it is of an acceptable scientific standard, however I have significant reservations, as outlined above.
